# Association of adiponectin gene expression with atrial fibrillation in a Pakistani populace

**DOI:** 10.1038/s41598-023-46388-2

**Published:** 2023-12-18

**Authors:** Saira Rafaqat, Saima Sharif, Mona Majeed, Shagufta Naz, Muhammad Saqib, Farkhanda Manzoor

**Affiliations:** 1 Department of Zoology, Lahore College for Women University, Lahore, Pakistan; 2 Emergency Department, Punjab Institute of Cardiology, Lahore, Pakistan; 3 Department of Medicine, Sir Ganga Ram Hospital, Lahore, Pakistan

**Keywords:** Molecular biology, Cardiology, Endocrinology

## Abstract

Adiponectin, an adipocytokine produced and secreted by adipose tissue, has anti-diabetic, anti-atherogenic, and anti-inflammatory properties. This case-control study was aimed to assess the expression and serum levels of adiponectin in subject suffereing from atrial fibrillation (AF). The study's subjects (n = 690) were enrolled from the Punjab Institute of Cardiology, Lahore and were grouped into control, AF without Metabolic syndrome (MetS), and AF with MetS groups. Along with the collection of demographic data, an analysis of adiponectin and biochemical parameters were performed. A highly significant difference in serum levels of adiponectin was observed among the control, AF without MetS, and AF with MetS groups (61.61 ± 45.30 ng/ml, 37.20 ± 19.46 ng/ml, 63.78 ± 61.69 ng/ml). The expression analysis of adiponectin was decreased (n-fold = ~ 0.30) in AF without MetS group as compared to control group (n-fold =  ~ 1.16) but increased in AF with MetS group (n-fold = ~ 6.26). The correlation analysis revealed a highly significant positive relationship between the expression of the *adiponectin* gene with waist-to-hip ratio (WHR) in AF without MetS group. Whereas, serum adiponectin was negatively related to serum triglycerides (TG) in AF with MetS group. In multiple regression analysis using adiponectin expression as the dependent variable, WHR was a determinant in AF without MetS. Whereas, when serum adiponectin was used as the dependent variable, serum TG was the determinant in group AF with MetS. The present study implicates that decreased expression and serum levels of adiponectin were associated with the development of AF in which WHR and serum TG also contributed towards the onset of atrial fibrillation.

## Introduction

The most prevalent heart arrhythmia and a severe condition affecting global public health is atrial fibrillation (AF). It was linked to more deaths, systemic thrombosis, and cardiac failure. It increases mortality and disease rates globally. According to future predictions, the absolute burden of atrial fibrillation could increase by more than 60% by 2050^1^. The mechanisms underlying AF are complicated and still not fully understood. Research has focused on the connection between inflammatory adipocytokines and AF as an outcome of data that has been available in the last ten years revealing that AF is connected to inflammation and obesity^2^. Adiponectin is an adipocytokine produced and released by adipose tissue that has anti-inflammatory, anti-diabetic, and anti-atherogenic effects^3^. Recent research has focused significantly on adiponectin, the most common adipocytokine, due to its relationship to inflammation and obesity-related cardiovascular disease^4^.

The occurrence of AF was independently correlated with adiponectin levels^5^. Likewise, paroxysmal AF patients < 65 years old with greater levels of adiponectin may be more at risk for recurrent AF following catheter ablation, according to earlier prospective studies^4^. Lower circulating adiponectin, on the other hand, was associated with a higher risk of severe cardiovascular events after anticoagulation in women with AF but not in males^6^. To have metabolic syndrome (MetS), at least three of the next five diseases must be present. These conditions include central obesity, high blood pressure, elevated triglycerides in the blood, low HDL levels, and elevated blood sugar^7^. The relationship between atrial fibrillation with MetS has been reported^8^. The high prevalence of overweight and obesity was present in the Pakistani adult population^9^. Epidemiological studies have provided increasing evidence that obesity is a significant risk factor for AF^10^ However, the primary pathophysiological processes underlying this relationship remain unknown.

Adiponectin plays a crucial part in numerous processes, including energy metabolism, inflammation, and cell proliferation, and is also a biomarker of metabolic syndrome. As a result, the link between serum adiponectin and AF has been documented^11^. Existing literature has examined serum adiponectin levels in various populations with atrial fibrillation, yet this aspect has not been explored within the Pakistani population. The novel aspect of this study is the evaluation of the gene expression of adiponectin in atrial fibrillation, a gap that has yet to be addressed in the literature. Consequently, this case control study aimed to assess the expression and serum levels of adiponectin in atrial fibrillation.

## Results

### Baseline demographics, Risk factors and comorbidities of subjects

The pattern of doing regular exercise, alcohol consumption, smoking status, history of diabetes mellitus, family history of AF, hypertension, and sleep pattern were significantly differ among the studied groups  (Table 1). The mean ± SD values of studied variables i-e age, SBP, DBP, heart rate, BMI, WHR, FBG, TG and TSH were presented in (Table 2). A highly significant difference (*p* ≤ 0.01) in mean values were observed among the control group, AF without MetS and AF with MetS subjects regarding SBP, DBP, heart rate, BMI, WHR, FBG, TG, and TSH (Table 2).

**Table 1:** Baseline demographics, risk factors and comorbidities in studied groups.

Variables	Control group (n = 230)	AF without MetS (n = 230)	AF with MetS (n = 230)	*p*-value
Gender, n (%)				
Male	108 (47)	120 (52)	106 (70)	0.369
Female	122 (53)	110 (48)	124 (54)	
Regularly exercise, n (%)				
NO	187 (81)	226 (98)	201 (87)	0.001**
Yes	43 (19)	04 (1.7)	29 (13)	
Alcohol consumption, n (%)				
NO	230 (100)	214 (93)	217 (94)	0.001**
Yes	–	16 (7)	13 (5.6)	
Smoking status, n (%)				
No	212 (92)	186 (81)	202 (87)	0.001**
Yes	18 (8)	44 (19)	28 (12)	
History of diabetes mellitus, n (%)				
NO	210 (91)	222 (97)	141 (61)	0.001**
Yes	20 (09)	08 (3.4)	89 (39)	
Family history of AF, n (%)				
NO	204 (87)	210 (91)	173 (75)	0.001**
Yes	26(11)	20 (8.6)	57 (25)	
Hypertension, n (%)				
NO	230 (100)	230 (100)	04 (1.7)	0.001**
Yes	–	–	226 (98)	
Continuous sleepers, n (%)				
NO	–	134 (58)	162 (70)	0.001**
Yes	230 (100)	96 (42)	68 (30)	
Intermittent sleepers, n (%)				
NO	230 (100)	97 (42)	74 (32)	0.001**
Yes	–	133 (48)	156 (66)	

Categorical variables are presented as numbers (percentage), and *p* values < 0.01 are indicated in bold.

*AF* atrial fibrillation, *MetS* metabolic syndrome.

**Table 2:** Clinical characteristics of control, AF without MetS and AF with MetS subjects.

Clinical characteristics	Control group (n = 230)	AF without MetS (n = 230)	AF with MetS (n = 230)
Age (years)	57.86 ± 11.32	58.40 ± 11.23	58.40 ± 11.23
SBP (mmHg)	112.43 ± 8.10	126.45 ± 21.42	134.30 ± 36.52**
DBP (mmHg)	85.34 ± 5.80	83.22 ± 13.48	93.04 ± 18.07**
Heart rate *(*bpm)	68.08 ± 6.79	121.45 ± 30.60	116.80 ± 30.83**
BMI (kg/m^2^^2^)	23.37 ± 2.12	29.28 ± 5.55	28.22 ± 10.33**
WHR	0.81 ± 0.05	0.80 ± 0.05	0.93 ± 0.05**
FBG (mg/dl)	89.59 ± 8.76	122.39 ± 38.56	140.77 ± 50.15**
Serum TG (mg/dl)	84.14 ± 66.169	135.01 ± 68.13	156.94 ± 55.99**
Serum TSH (uIU/ml)	1.96 ± 1.08	1.41 ± 1.15	1.69 ± 2.00**
Serum adiponectin (ng/ml)	61.61 ± 45.30	37.20 ± 1946	63.78 ± 61.69**
Expression of *adiponectin* gene (Arbitrary units)	1.16 fold	0.30 fold	6.26 fold**

Continuous variables are presented as mean ± SD by ANOVA.

*P* < 0.01** is considered a highly significant difference among the groups.

*AF* atrial fibrillation, *MetS* metabolic syndrome, *SBP* systolic blood pressure, *DBP* diastolic blood pressure, *bpm* beats per minute, *BMI* body mass index, *WHR* Waist-Hip Ratio, *FBG* fasting blood glucose, *TG* triglycerides, *TSH* thyroid stimulating hormone.

### Assessment of serum levels of adiponectin and expression of adiponectin

The serum level of adiponectin differ significantly in control group, AF without MetS group and AF with MetS group (61.61 ± 45.30 ng/ml, 37.20 ± 19.46 ng/ml, 63.78 ± 6169 ng/ml). The relative expression of the adiponectin gene was quantitatively expressed as an n-fold difference relative to the reference gene (GAPDH).

A significant difference was observed in the expression profile of adiponectin gene, which was elevated by 6.26-fold in the AF with MetS group compared to 0.30-fold in the AF without MetS group and 1.16-fold in control group (Table 2).

### Pearson correlation analysis

When Pearson correlation was applied to find the relationship between the serum and expression of adiponectin with clinical parameters of AF and MetS such as age, SBP, DBP, heart rate, BMI, WHR, FBG, TG and TSH. It was observed that the expression of the adiponectin gene was directly correlated to WHR (r = 0.205, p = 0.002) in AF without MetS group. However, a negative relationship was observed between serum adiponectin with serum TG (r = − 0.182, *p* = 0.006) in AF with MetS group (Table 3).

**Table 3:** Correlation analysis of serum levels and expression of adiponectin with clinical parameters of AF and MetS in studied groups.

Clinical parameters	*r*-value of expression *adiponectin*			*r*-value of serum Adiponectin		
	Control group	AF without MetS	AF with MetS	Control group	AF without MetS	AF with MetS
Age (years)	− 0.025	0.072	− 0.039	0.109	0.073	− 0.007
SBP (mmHg)	− 0.019	0.049	− 0.103	− 0.024	0.041	0.049
DBP (mmHg)	− 0.011	− 0.019	− 0.055	0.006	0.032	− 0.039
Heart rate (bpm)	− 0.049	− 0.004	− 0.112	0.050	− 0.004	0.016
BMI (kg/m^2^)	0.075	− 0.061	− 0.052	0.003	0.026	− 0.025
WHR	− 0.031	0.205**	− 0.004	− 0.008	− 0.075	− 0.004
FBG (mg/dl)	− 0.079	0.001	− 0.082	− 0.043	− 0.009	− 0.004
TG (mg/dl)	− 0.091	0.056	0.052	− 0.014	− 0.064	− 0.182**
TSH (uIU/ml)	− 0.004	− 0.097	− 0.066	0.048	− 0.029	− 0.013

Bivariate Pearson correlation analysis was performed.

** Correlation is significant at 0.01 level (two-tailed).

*SBP* systolic blood pressure, *DBP* diastolic blood pressure, *BMI* body mass index, *WHR* waist-high ratio, *FBG* fasting blood glucose, *TG* triglycerides, *TSH* thyroid stimulating hormone.

### Stepwise linear regression analysis

Stepwise linear regression was carried out considering serum levels and expression of adiponectin as dependent variables and age, DBP, SBP, heart rate, BMI, WHR, FBG, TG and TSH as independent variables. It was observed that no models were computed in control group in either case.

In AF without MetS group one model was computed showing WHR (β = 0.201; *p* = 0.002) as an important determinant of AF (Table 4), Whereas one model was computed in AF with MetS group showing serum TG (β = − 0.182, *p* = 0.006) as important determinant of the disease (Table 4).

**Table 4:** Stepwise linear regression of AF without MetS and AF with MetS groups.

Variable	*B*	95% CI		*SE B*	β	*P*-value	*R*^2^	Δ*R*^2^	Sig. F Change
		LL	UL						
AF without MetS group (Expression of *adiponectin* gene)									
Model 1							0.042	0.042	0.002
(Constant)	− 2.346	− 4.001	− 0.690	0.840		0.006			
WHR	3.280	1.237	5.323	1.037	0.205	0.002			
AF with MetS group (Serum levels of adiponectin)									
Model 1							0.033	0.033	0 .006
(Constant)	95.260	71.708	118.812	11.953		0.001			
Serum TG	− 0.201	− 0.342	− 0.059	0.072	− 0.182	0.006			

Analysis of data was done using stepwise linear regression. *WHR* Waist-Hip Ratio, *TG* triglycerides.

## Discussion

The goal of the study is to analyze the serum and expression levels of adiponectin in AF subjects. As a result, expression and serum levels of adiponectin were associated with the development of AF in which WHR and serum TG also contributed towards the onset of atrial fibrillation. Adipokines, also known as adipocytokines, are cell-signalling molecules (cytokines) generated by adipose tissue that have an impact on the body's energy/metabolic state, inflammation, obesity, etc. Leptin, adiponectin, resistin, interleukin-6, and tumor necrosis factor are a few prominent adipokines^12^. Among them, we focused on adiponectin because of its following roles in AF. (i) Adiponectin may be connected to atrial remodelling, autonomic dysfunction, and inflammation as pathogenetic factors for AF. (ii) Adiponectin might serve as a link between fat tissue and the heart, influencing cardiac remodelling. It may be a real modulator of the link between fat and heart remodelling by controlling changes in blood pressure and cardiac output or affecting myocardial alterations^13^. (iii) By its effects on endothelial function, atherogenesis, and vascular inflammation, adiponectin plays a role in cardiac remodelling in addition to its direct effects on the heart muscle. Adiponectin also crosses the blood–brain barrier, influencing the heart's function through the central nervous system^14^.

According to various studies, serum adiponectin played a significant role in the pathogenesis of AF but very little literature was accessible in the Pakistani population and expression of adiponectin was not reported in AF subjects. Therefore, adiponectin seems to be more specific as compared to other adipokines.

In the current research, it was observed that adiponectin expression and serum levels were substantially lower in AF patients. This finding may be related to the fact that adiponectin levels may be reduced by inflammation, calcium channel overload, and ectopic focal activities. In this study the correlation analysis, reavealed a strong positive connection between the activity of the adiponectin gene and waist-to-hip ratio. Conversely, among those with AF and MetS, a negative correlation between serum adiponectin levels and serum triglyceride levels was observed. Additionally, stepwise linear regression analysis determined that the expression of the adiponectin gene was linked with WHR in AF subjects whereas the serum levels of adiponectin were associated with serum triglyceride levels in individuals with AF subjects suffering from metabolic syndrome.

In the studied groups, the participants were of similar age. Wang et al. reported that adiponectin receptors may become downregulated or resistant to ageing, which can produce positive feedback^15^. Although older people tend to have higher levels of adiponectin, the prevalence of AF progressively rises with age, thus the raised levels may not have the same positive benefits^16^. Age is the main risk factor as well as the cause of AF. The prevalence of AF considerably increased with age in adult and elderly European men and women. However, there is a lack of study on how the risk variables affect the age at AF onset^17^.

In this study, SBP and DBP were increased in AF with MetS groups as compared to the control group and AF without MetS group. A key component of metabolic syndrome is elevated blood pressure. Even in the absence of diabetes, high blood pressure or hypertension was present in more than 85% of persons with metabolic syndrome^18^. The risk of AF eventually increased by hypertension, which also causes more cases of AF than any other risk factor due to its great prevalence in the population. Between 60 and 80% of patients with established AF have hypertension. The pathogenetic processes behind the higher propensity of hypertensive individuals to develop AF was still poorly understood, despite the epidemiological link between hypertension and AF being well-established^19^.

A highly significant difference in BMI and WHR was noticed in the studied groups. Correlation analysis and stepwise regression also indicated that positive relationship between WHR with gene expression of adiponectin in AF subjects. This suggests that individuals with a more central fat distribution might experience a downregulation of the adiponectin gene, which could contribute to metabolic dysregulation and increased risk of conditions like insulin resistance, type 2 diabetes, and cardiovascular diseases. Conversely, individuals with a lower WHR, indicating a healthier distribution of fat, tend to have better adiponectin gene expression levels. This can be associated with improved metabolic health and a decreased risk of the aforementioned conditions. It's important to note that while there was a correlation between WHR and adiponectin gene expression. Multiple factors, including genetics, lifestyle, and overall health, can influence this relationship. BMI has been recommended as the best indicator of adiposity since it is simple to calculate and has a strong relationship with the health risks associated with obesity^15^. High BMI is a significant indicator of the occurrence and development of AF^20^. Compared to non-obese individuals, obese people have a chance of getting AF which is almost 50% higher. Despite the lack of clarity surrounding the fundamental pathophysiological mechanisms, numerous laboratory and clinical studies have linked obesity to the development and persistence of atrial fibrillation^21^.

An increase in body mass, as assessed through BMI, was linked to an elevated likelihood of experiencing new-onset AF. A distribution of excess abdominal fat, as quantified by WHR, was connected to a higher probability of developing new-onset AF in males, while this correlation was not observed in females^22^. Obesity triggers changes in the structure of the atria, a significant process in the development of AF^23^. This remodeling of the atria can be instigated by metabolic disturbances, a mild inflammatory state, and increased pressure within the atria due to various substances secreted by visceral fat, both locally and systemically^24–26^. Atrial remodeling can also be prompted by accompanying conditions like hypertension and metabolic irregularities^25^.

In this research, AF group with MetS were found to have higher levels of FBG than AF group without MetS and the control group. Diabetes mellitus was also reported to be a risk factor for AF. According to Chao et al. (2010) abnormal glucose metabolism changes the characteristics of the biatrial substrate, resulting in intra-atrial conduction delays, reduced voltage, and a higher return rate after catheter ablation^27^.

In this study elevated serum TG levels in AF with MetS group as compared to AF without MetS and the control group. Correlation analysis as well as stepwise linear regression indicated that serum triglycerides were negatively associated with serum adiponectin levels in AF subjects suffering from metabolic syndrome. Numerous research studies have explored the connection between adiponectin and levels of lipids in the bloodstream^28–33^. Similarly, Li et al. (2018) observed that AF patients had significantly higher levels of TG^34^. The paradoxical inverse connection between lipid levels and the underlying causes of atrial fibrillation, along with its clinical significance, remains a mystery^35^. Hypertriglyceridemia was strongly associated with all components of metabolic syndrome^36^.

In the context of metabolic syndrome, the relationship between serum adiponectin levels and serum triglycerides follows an inverse pattern. Individuals with metabolic syndrome often experience lower levels of adiponectin in their bloodstream. Adiponectin is a hormone that plays a role in regulating various metabolic processes, including lipid metabolism. The inverse relationship between serum adiponectin levels and serum triglycerides in metabolic syndrome suggests that as adiponectin levels decrease, triglyceride levels tend to increase. Adiponectin is known to have anti-inflammatory and insulin-sensitizing effects. When its levels are reduced, it can contribute to metabolic dysfunction, insulin resistance, and dysregulation of lipid metabolism, leading to elevated triglyceride levels. Conversely, higher adiponectin levels are associated with improved lipid profiles and better overall metabolic health. Therefore, in the context of metabolic syndrome, the lower adiponectin levels might contribute to the elevated triglyceride levels commonly observed in individuals with this condition. It was observed the concentration of the adiponectin hormone was linked with a negative association with low-density lipoprotein and serum triglycerides, as well as a positive correlation with high-density lipoprotein^37–39^.

Decreased levels of TSH in AF without MetS and AF with MetS groups as compared to the control group were observed in this study The formation of atrial arrhythmogenesis, which leads to the onset of AF, is exacerbated by hypothyroidism in both animal models and human populations. Following catheter therapy for AF, high-normal TSH levels may independently indicate the return of atrial tachyarrhythmias in addition to hypothyroidism^40^. Another study reported a positive correlation between TSH levels and the onset of MetSas well as weight gain^41^.

In the present study, the expression and serum levels of adiponectin in the Pakistani population were elevated in AF with MetS group as compared to control group and AF without MetS group. Adiponectin levels in individuals with persistent AF were found to be greater than levels of a marker for collagen type 1 degradation which might be due to the stimulation of fibrosis, which manifests as fibrotic thickening and fibroblasts, also results in atrial structural remodeling^29,42,43^. According to Zhu et al. (2021), AF patients and controls with normal sinus rhythm revealed that the serum adiponectin levels in AF patients were considerably higher than those in controls with normal sinus rhythm, It was discovered that indicators of heart remodelling, inflammation, and cardiac autonomic function are all associated with serum adiponectin^16^.

Although the underlying processes are not fully known. According to Shimano et al. a disconnection between adiponectin and the adiponectin receptors in AF may be the reason for the elevated levels of adiponectin, which would then cause an increase in adiponectin release^42^. Overproduction by the muscle tissue is another reason for pathologically elevated levels of adiponectin. Earlier studies have shown that skeletal muscle has enhanced expression of adiponectin in the presence of mild-to-moderate heart failure, along with concomitant adiponectin resistance^44^. Adiponectin and AF risk also continue to have an uncertain connection^45^.

The expression and serum levels of adiponectin were also found to be lower in AF without MetS in this study. A substantial correlation between paroxysmal AF and a plasma adiponectin concentration that was comparatively low was found^46^. Furthermore, Hernandez-Romero et al. found a link between AF patients' reduced adiponectin levels and poor cardiovascular outcomes^6^. Patients with cardiovascular disease had lower adiponectin levels, which was consistent with its preventive effect^47^. It is well known that adiponectin levels are lower in the male sex, and it has been hypothesized that testosterone reduces adiponectin synthesis^48^. However, there has been much debate about the potential contradictory function of adiponectin. Low levels of adiponectin among low-risk individuals who have a normal inflammatory response have been proposed as a marker for cardiovascular risk, whereas high levels of adiponectin among individuals at high risk seem to play a compensatory role against inflammation, vascular remodelling, and endothelial damage^49^.

Several factors play a role in the aetiology of AF. Stretching and inflammation increase angiotensin II levels, leading to calcium excess and aberrant focal activity which initiates AF^50,51^. The duration of AF may be supported by structural alterations to the atria and inflammation. In addition to adiponectin level, another study demonstrated that left atrial (LA) size is a factor in AF. This suggests that adiponectin and LA size, which represent pathophysiological alterations, may work together to accelerate the progression of AF. The high prevalence of AF following cardiac surgery (25–40%) led to the early hypothesis that inflammation may have a role in at least certain types of AF. Although the exact mechanisms were not completely understood, it was reported that lower levels of adiponectin in AF patients may be linked to its anti-inflammatory properties^46^.

## Materials and methods

 This case–control study was conducted in Lahore College for Women University, Lahore. The non-probability consecutive sampling method was adopted to enroll the participants at the Punjab Institute of Cardiology, Lahore, Pakistan from July 2021 to December 2022.

The physicians made the diagnosis of AF in the study’s participants after analyzing their ECGs, which showed irregular R-R intervals caused by irregular impulse conduction to the ventricles, no P waves, and disorganized electrical activity in their atria. This study was approved by the ethical review committee (ref.no: RTPGME-Research-179) of the Punjab Institute of Cardiology, Lahore, Pakistan and LCWU (No. REF/NO/LCWU/ZOO/690-; Dated: 01-01-2021) Lahore. All patients were enrolled after their consent for participation in the study.

The sample size was calculated by the Rao software which was based on the prevalence of the disease and with a margin of error of 5%. 230 healthy individuals (control group) without a history of AF, diabetes, or hypertension, 230 individuals with AF but no MetS, and 230 individuals with AF and MetS were selected, froming a total of 690 potential participants for this research.

### Inclusion criteria of AF subjects

The inclusion criteria of AF subjects was t age (≥ 18 years), both sexes (males and females), subjects with a previous history of stroke, coronary artery disease, myocardial infarction, transient ischemic attack, prior coronary artery bypass graft surgery, systemic embolism or percutaneous coronary intervention were enrolled in AF without MetS group. AF Subjects suffering from diabetes mellitus with hypertension or taking medications with elevated fasting blood glucose levels were included in the AF with MetS group. The criteria used for Metabolic syndrome was National Cholesterol Education Program Adult Treatment Panel III (NCEP ATP III) (≥ 3) in which waist circumference for males > 40 inches, females > 35 inches, Low high-density lipoprotein cholesterol (men < 40 mg/dl, women < 50 mg/dl), elevated triglyceride (triglyceride ≥ 150 mg/dl), or hypertension (systolic blood pressure ≥ 130 or diastolic blood pressure ≥ 85 mm Hg), hyperglycemia (fasting blood glucose ≥ 100 mg/dl)^11^. When we assigned the patients with Metabolic Syndrome, we deliberately focused on three distinct criteria (systolic blood pressure over 130 or diastolic blood pressure over 85 mm Hg for hypertension, fasting blood glucose equal to or greater than 100 mg/dl, and waist circumference exceeding 40 inches for males or 35 inches for females). These parameters were assessed during the scrutinizing of the subjects.

### Exclusion criteria of AF subjects

Subjects who planned to undergo pulmonary vein ablation or surgery to treat atrial fibrillation were excluded from the research, as were women who were pregnant.

### Assessment of demographic, risk factor and comorbidities data

The subjects were enrolled after taking their content and a questionnaire was filled out which included information regarding age (years), gender (male and female), pattern of doing exercise, alcohol consumption status, history of diabetes mellitus, hypertension, family history of AF, sleep patterns (continuous sleepers and intermittent sleepers), body weight in kilograms, height in centimetres without shoes and blood pressure were also noted.

### Measurements of blood pressure, BMI and WHR

The sphygmomanometer was used to measure both the systolic and diastolic blood pressure. The hospital’s medically trained staff supervised the process. The body mass index (BMI) was calculated by using the formula^52^.

$${\text{BMI}} = {{{\text{Weight }}\left( {{\text{kg}}} \right)} \mathord{\left/ {\vphantom {{{\text{Weight }}\left( {{\text{kg}}} \right)} {\left( {{\text{Height}}\left( {\text{m}} \right)} \right)^{2} }}} \right. \kern-\nulldelimiterspace} {\left( {{\text{Height}}\left( {\text{m}} \right)} \right)^{2} }}$$

The waist-to-hip ratio was calculated by using the formula^52^.

$${\text{WHR}} = {{{\text{Waist }}\left( {{\text{cm}}} \right)} \mathord{\left/ {\vphantom {{{\text{Waist }}\left( {{\text{cm}}} \right)} {{\text{Hip }}({\text{cm}})}}} \right. \kern-\nulldelimiterspace} {{\text{Hip }}({\text{cm}})}}$$

### Collection of blood sample

The blood samples were collected from median cubital vein. The collected blood samples were transferred into the serum separation tubes for collection of serum and sterile EDTA-coated tubes for separation of  RNA. After 30 min, the serum was obtained by centrifuging the serum separation container at 3000 rpm for 15 min to obtain the serum. The serum samples were kept until analysis at -80 °C. The obtained serum was used for analysis of triglycerides, TSH and serum adiponectin.

### Biochemical assessments

The fasting blood glucose was measured by a glucometer. The serum triglycerides (TG) were measured on a chemistry analyzer (ERBA Chemistry Analyzer, Model # CHEM-7, Serial # 9047) using the biochemical kit in the research laboratory of LCWU, Lahore.

### Measurements of adiponectin and TSH levels

The serum levels of adiponectin and TSH were determined using adiponectin and TSH human ELISA kits from Wuhan Fine Biotech Co., Ltd. (Cat. # EH2593 and Cat. # EH0401 respectively). Following the formation of a standard curve using the absorption values of the standard solutions, serum levels were calculated using the standard curve (Biotek Elx800 Microplate Reader).

### RNA isolation and cDNA synthesis

Blood samples collected in EDTA tubes were processed for mRNA extraction within 2–4 h of collection, and mRNA was extracted using the Trizol technique^53^ (Refrigerated Centrifuge Machine HARRIER 18/80, UK). We evaluated the quantity and purity of mRNA using Nanodrop (Multiskan SkyHigh Microplate spectrophotometer, UK). Thermo Scientific RevertAid First Strand cDNA Synthesis Kit (cat # K1622) was used to transform mRNA into cDNA for the purpose to study gene expression (Programmable Thermal Cycler Ptc-06 UK). Gel electrophoresis was used for the cDNA validation.

### Expression analysis by real-time PCR

Oligonucleotide primers were created using Primer 3 software to conduct the Real-Time PCR (Applied Biosystems Step One ™ Real-Time PCR instrument, Thermo Scientific Fisher Inc. US). The primer sequence and optimization conditions were shown in (Table 5). An easily accessible industry produced the primer. Maxima SYBER Green/ROX qPCR Master Mix from Thermo Scientific (CAT # k0221) was used to assess the relative expression of adiponectin gene. To regulate the target gene's expression, the Glyceraldehyde 3-phosphate dehydrogenase (GAPDH) gene was used as a standard. The RT-PCR conditions consisted of a single cycle of 94 °C for 4 min, followed by 30 cycles of 94 °C for 30 s, 59 °C for 20–30 s, and 72 °C for 45 s. It took five minutes at 72 °C for the ultimate expansion.

**Table 5:** Designed primer used in the study.

Gene	Primers	Product size (bp)	TM (°C)
GAPDH	F: ATCCCATCACCATCTTCCAGGA	122	59
	R: CAAATGAGCCCCAGCCTTCT		
*Adiponectin*	F: CATGACCAGGAAACCACGACT	301	59
	R: TGAATGCTGAGCGGTAT		

### Statistical analysis

SPSS version 22.0 software was used for this research. To analyze the categorical data, the Chi-square test was employed. The control group, the AF without MetS, and the AF with MetS groups' mean values were compared using a set of analysis of variance (ANOVA) analyses. The expression and serum levels of adiponectin were correlated with clinical parameters of AF and MetS such as age, SBP, DBP, heart rate, BMI, WHR, FBG, TG, and TSH using a bivariate Pearson correlation analysis. Stepwise multiple regression was done to study the effect of serum and expression of adiponectin on the clinical parameters of AF and MetS. Data of expression of genes was expressed as a fold change and relative gene expression levels were assessed using comparative CT value (2 − ΔΔCT). The level of significance was considered at 95% and 99% of the probability level.

### Ethical approval

The study was conducted according to the guidelines of the Declaration of Helsinki, and approved by the Institutional Ethical Review Committee of Lahore College for Women University, Lahore (Ref/No/LCWU/ZOO/690) ﻿and by the ethical review committee of Punjab Institute of Cardiology, Lahore, Pakistan. (Ref.No: RTPGME-Research-179). All authors meet the International Committee of Medical Journal Editors (ICMJE) criteria for authorship for this article, take responsibility for the integrity of the work as a whole, and have given their approval for this version to be published.

### Consent to participate

Prior to their participation, all the participants gave their informed consent, and the data were either pseudonymized or anonymized, depending on the circumstance.

## Conclusion

It has been concluded in the present study that the serum levels and expression of adiponectin significantly decreased in AF subjects. This may be that decreased levels of adiponectin are the results of inflammation, calcium channel overload and ectopic focal activities. Whereas adiponectin expression and serum levels were increased in the group suffering from AF with MetS, which might be due to the excess adipose tissue which might lead to cardiac remodeling which could alternate the electrical conduction system in AF. Large-scale prospective studies should be used to verify these findings, and subsequent research should focus on determining the mechanisms underlying the link between adiponectin and atrial fibrillation.

## Data Availability

The data that support the findings of this study are available on request from the corresponding author [Prof. Dr Saima Sharif].
